# Empowering Women’s Health: A Global Perspective on Artificial Intelligence and Robotics

**DOI:** 10.7759/cureus.49611

**Published:** 2023-11-28

**Authors:** Munaza Afaq, Divya E Abraham, Saloni H Patel, Areen D Al-Dhoon, Zara Arshad

**Affiliations:** 1 Internal Medicine, Global Remote Research Scholars Program, Saint Paul, USA; 2 Neurology, Atrium Health Wake Forest Baptist Medical Center, Winston-Salem, USA; 3 Internal Medicine, Bolan University of Medical and Health Sciences, Quetta, PAK; 4 Clinical Research, Shifa International Hospital Islamabad, Islamabad, PAK

**Keywords:** challenges to health innovation, health-care equity, international collaboration and development, maternal mortality, global health policy, women’s health, artificial intelligence and robotics in healthcare, global health and artificial intelligence

## Abstract

In our rapidly evolving world, technology stands at the forefront, driving remarkable advancements across various sectors. One of the most notable changes is the use of Artificial Intelligence (AI) and robotics in healthcare, starting a revolution that has the power to change women's health all over the world. Developed nations are already witnessing the benefits. However, a significant portion of the global population in underdeveloped regions is lagging behind, resulting in a noticeable disparity. This is particularly evident in women’s healthcare, an area already facing global inequities.

As we witness a digital revolution, we examine the progressive steps taken in women's healthcare. AI and robotics are key to this transformation. The services range from using data to predict cancer trends to tailor-made medicine and technologies in reproduction. This editorial addresses the existing gaps and the digital divide, exploring the necessity for an inclusive approach in technology design and implementation to ensure equitable healthcare access.

Furthermore, it highlights the imperative role of multi-sectoral collaborations to foster innovation while mitigating risks. The clear goal is to build a future where all women, no matter where they live, can get good healthcare, helped by AI and robotics, bringing in a time of healthcare for all. It's crucial for everyone involved to come together to make a healthcare system that everyone can use, helping women everywhere with the help of new technology.

## Editorial

Artificial Intelligence (AI) and robotics have ushered in a new era of innovation in women's healthcare. The strides made in the early identification of cervical cancer are truly remarkable. Through the application of AI in colposcopy and magnetic resonance imaging, the diagnosis and staging of cervical cancer have seen notable enhancements, showcasing encouraging results [1]. AI algorithms have demonstrated considerable potential in advancing the early detection and diagnosis of breast cancer. Leveraging deep learning and pattern recognition, these algorithms can analyze medical imaging, such as mammograms, with a high degree of accuracy, often equal to or even surpassing that of human experts [2]. The capabilities of AI extend far beyond disease detection, reaching deeply into the essential area of reproductive health. It is actively reshaping the success rates of in-vitro fertilization (IVF) treatments through dynamic optimization techniques. By analyzing a wealth of data, AI can help identify the most opportune moments for embryo implantation, potentially improving the chances of successful pregnancies [3].

In recent years, AI has shown promising results in prenatal care, facilitating more accurate disease diagnosis, treatment planning, and patient monitoring, thereby enhancing maternal and fetal health outcomes. Utilizing vast datasets, AI models are capable of identifying individuals at high risk of adverse outcomes, assisting healthcare providers in making timely and precise decisions. Recent studies have underscored the potential of AI in predicting complications such as preterm birth, preeclampsia, and gestational diabetes using various health indicators and AI models [4]. Moreover, a substantial percentage of pregnant women are open to integrating AI into maternal healthcare, although acceptance levels are influenced by their education and familiarity with AI technology. This shift suggests a promising role of AI in advancing maternal health care [4].

Furthermore, the incorporation of robotics has significantly transformed the field, remarkably improving surgical accuracy, medical imaging, and reproductive technologies. The educational landscape for medical professionals has undergone a paradigm shift, with the inception of virtual reality (VR) platforms offering laparoscopic and robotic surgical simulations, a step that promises to hone skills to an unprecedented level of accuracy and competence. Telemedicine and augmented reality promise further enhancements, paving the way for even more comprehensive and accessible healthcare solutions. Envision a future where robots assist in childbirth and wearable devices monitor health parameters for high-risk pregnancies, ensuring timely intervention. A comforting virtual companion in the form of AI chatbots and sophisticated language models like ChatGPT [5] stands ready to guide and support new mothers through their journey. 

Despite all these advances, the sad truth remains while rich countries move forward quickly, resource-limited countries struggle to keep up. The challenges are multifaceted. The huge expenses tied to the newest AI and robotic systems stand as substantial hurdles, not to mention the necessity of channeling resources towards more pressing health needs such as battling infectious diseases and improving healthcare access. Infrastructure is often insufficient, with recent reports highlighting a substantial disparity in electricity and internet availability in developing regions [6,7]. This is further complicated by issues surrounding data accessibility, privacy, and security. Addressing these prevalent challenges necessitates a comprehensive approach, with a need to design cost-effective AI and robotic solutions. A synergistic effort between tech industries, governmental bodies, and NGOs can help establish training initiatives, equipping local health professionals to handle and sustain these innovative technologies proficiently while also respecting cultural and societal intricacies. 

Low- and lower-income countries persist in accounting for the majority of maternal deaths. This underscores the urgent need to tackle the critical issue of maternal mortality. By providing remote prenatal consultations, telemedicine, backed by AI technology, can offer a lifeline in such areas. The approach can potentially reduce the number of unmonitored pregnancies and promote healthier pregnancies, leading to positive outcomes. Affordable and quick disease screening is possible through AI-powered diagnostic tools that efficiently process medical imagery. AI-powered chatbots, capable of educating in local languages, can improve health literacy concerning reproductive and sexual health. AI also can optimize patient data management, giving an opportunity to understand and address the unique healthcare needs of women in a specific region. Remote-operated robotics stands to redefine gynecology. Establishing comprehensive training programs to empower healthcare workers in distant locations with the expertise to utilize these advanced technologies could spark a wave of positive change. AI and robotic systems can create immersive training modules, including virtual tutorials and diagnostic simulations. Tailoring solutions to resonate with the diverse cultural intricacies can facilitate a smoother integration of technology into the existing healthcare paradigms.

While we marvel at the spectacular advancements AI and robotics bring to women's healthcare, we must remain grounded. The real triumph will not lie in the sophistication of the technology but in ensuring its benefits reach every corner of the globe, eventually helping attain universal health coverage, a goal we are too far from achieving. The journey ahead is difficult, but the potential benefits are too great to ignore. The aspiration is to establish a network of healthcare where technology serves as a bridge connecting women globally, facilitating a canvas of health and well-being that is both inclusive and expansive. The challenge is set; the question remains: can we rise to meet it?
